# Sounding out falsified medicines from genuine medicines using Broadband Acoustic Resonance Dissolution Spectroscopy (BARDS)

**DOI:** 10.1038/s41598-021-90323-2

**Published:** 2021-06-16

**Authors:** Anas Alfarsi, Céline Caillet, Garry Fawbert, Simon Lawrence, Jacob Krüse, Seán McSweeney, Marcus O’Mahony, Arjen Dondorp, Paul N. Newton, Dara Fitzpatrick

**Affiliations:** 1 School of Chemistry, Analytical and Biological Chemistry Research Facility (ABCRF), University College Cork, Cork, Ireland; 2 Lao-Oxford-Mahosot Hospital-Wellcome Trust Research Unit, Microbiology Laboratory, Mahosot Hospital, Vientiane, Lao PDR; 3 WorldWide Antimalarial Resistance Network (WWARN) and Infectious Disease Data Observatory (IDDO), Centre for Tropical Medicine & Global Health, Nuffield Department of Medicine, University of Oxford, Oxford, UK; 4 Centre for Tropical Medicine & Global Health, Nuffield Department of Medicine, University of Oxford, Oxford, UK; 5 eGlaxoSmithKline Plc (GSK), Ware, UK; 6 Kinetox, Beilen, The Netherlands; 7 Cork Institute of Technology, Cork, Ireland; 8 Pharmaceutical Manufacturing Technology Centre, Bernal Institute, University of Limerick, Limerick, Ireland; 9 Mahidol Oxford Research Unit (MORU), Faculty of Tropical Medicine, Mahidol University, Bangkok, Thailand; 10 Present Address: Department of Chemistry, Faculty of Science, Albaha University, Albaha, Saudi Arabia

**Keywords:** Health care, Disease prevention, Techniques and instrumentation

## Abstract

The trade in falsified medicine has increased significantly and it is estimated that global falsified sales have reached $100 billion in 2020. The EU Falsified Medicines Directive states that falsified medicines do not only reach patients through illegal routes but also via the legal supply chain. Falsified medicines can contain harmful ingredients. They can also contain too little or too much active ingredient or no active ingredient at all. BARDS (Broadband Acoustic Resonance Dissolution Spectroscopy) harnesses an acoustic phenomenon associated with the dissolution of a sample (tablet or powder). The resulting acoustic spectrum is unique and intrinsic to the sample and can be used as an identifier or signature profile. BARDS was evaluated in this study to determine whether a product is falsified or genuine in a rapid manner and at lower cost than many existing technologies. A range of genuine and falsified medicines, including falsified antimalarial tablets from south-east Asia, were tested, and compared to their counterpart genuine products. Significant differences between genuine and falsified doses were found in their acoustic signatures as they disintegrate and dissolve. Principal component analysis was employed to differentiate between the genuine and falsified medicines. This demonstrates that the tablets and capsules included here have intrinsic acoustic signatures which could be used to screen the quality of medicines.

## Introduction

The increase in substandard and falsified (SF) pharmaceutical products is an increasing global issue. They affect, most adversely developing countries with an estimation of 10% of the market SF^1–5^. It has become increasingly clear, however, that it is a significant problem in developed countries also^6–9^.

Up until 2017 there was no consensus international definition of falsified medicines^10^. However, recently the World Health Organization defined substandard medical products as those authorized medical products that fail to meet either their quality standards or their specifications^3^. It defines unlicensed medical products as those which have not undergone evaluation or approval for the market in which they are distributed, subject to permitted conditions under national or regional regulation and legislation. Falsified medical products are defined as those products which deliberately misrepresent their identity, composition or source. Falsified medicines can include correct or incorrect ingredients, a lack of sufficient ingredients or falsified or misleading packaging^3^.

There has been a significant and dramatic apparent increase in the number of falsified medicines detected. In 2013, there were 2193 incidents of falsified medicines detected whereas in 2017, there were 3509 incidents identified by the Pharmaceutical Security Institute^5^. The WHO estimated that up to 169,000 young children die each year from pneumonia after treatment with substandard or falsified antibiotics^3^.

In Africa and Asia, substandard and falsified antimicrobial medicines represent a huge but neglected challenge to public health. The prevalence of falsified medicines has been documented in many studies^3^. Antimicrobial medicines have been the most extensively investigated class due to the significant prevalence of infectious diseases in these regions. Falsified lifestyle medicines such as phosphodiesterase type 5 (PDE-5) inhibitor drugs sildenafil citrate (Viagra), tadalafil (Cialis) and, more recently, vardenafil hydrochloride (Levitra), are very frequently falsified and detected globally^11^. This is largely due to the growth and ubiquitous use of the internet. Fraudulent websites have proliferated whereby any individual can easily and anonymously purchase prescription-only medicines; the purchaser is responsible for illegal purchase of these products.

The techniques used for detection and analysis of SF medicines are numerous. Gas chromatography, nuclear magnetic resonance, liquid chromatography (LC) coupled with mass spectrometry (MS) or UV-spectrophotometry detectors, colorimetry, thin layer chromatography, and capillary electrophoresis are all examples of these techniques^12,13^. Some, such as LC–MS or gas chromatography require laboratory environments and highly trained operators, whereas portable techniques based on innovative technology such as Raman and near-Infrared spectroscopy can be used for field screening of medicines as they are quick and require little or no sample preparation. These techniques range from Raman spectroscopy, X-ray powder diffraction, near infrared spectroscopy, to Fourier transform infrared spectroscopy^14^.

The significant costs involved in detecting and intercepting falsified and substandard medicines may be prohibitive, especially for countries with low health expenditure, making the implementation of systematic and global measures to combat this problem inadequate. The operation of apparatus and its continuing maintenance to ensure accurate results is another problematic issue. Therefore cost-effective, simple and reliable methods are vital interventions to protect these ‘soft-target’ countries where proliferation of falsified medicines is a problem.

BARDS is a simple dissolution based test that generates a unique acoustic spectrum for a given sample. It offers the ability to monitor and characterise the unique disintegration during dissolution of tablets and blends based on their acoustic spectrum when dissolved in a solvent^15^. Much effort has been targeted at improving the security features of packaging or embedding coatings with tracer materials^16^. However, the dissolution process of a tablet has an intrinsic acoustic profile and is a powerful signature of the product which falsifiers cannot yet mimic.

The unique acoustic profiles are due to changes in the compressibility of a solvent during dissolution which produces the BARDS signal. The speed of inducted sound in a vessel containing the solvent and formulation is reduced, resulting in frequency changes within the solution.

The speed of sound (*v*) in a solvent is determined by Eq. (1)

1 $${v}_{\left(sound\right)}=\sqrt{\frac{1}{K\rho }}$$

where ρ = mass density and *K* = compressibility, which is the inverse of the bulk modulus of the medium. Generation of gas bubbles in a liquid decreases the density in a negligible way in comparison to the large increase in compressibility. The net effect is a significant reduction of the sound velocity in the dissolution medium. Equation (2) demonstrates the relationship between the fractional gas volume and the speed of sound in water as derived by Frank Crawford^17^

2 $$\frac{{v}_{w}}{v}=\sqrt{(1+1.49x{10}^{4}{f}_{a})}$$

where *v*_*w*_ and *v* are the sound velocities in pure and bubble-filled water, respectively*. f*_*a*_ = the fractional volume occupied by air bubbles. The factor 1.49 × 10^4^ in Eq. (2) was calculated as shown in Eq. (3):

3 $${{(v}_{w})}^{2}{\rho }_{w} \frac{1}{ \gamma p}=1.49\times {10}^{4}$$

where *ρ*_*w*_ = the density of water, γ = ratio of specific heats for dry air and normal air and *p* = atmospheric air pressure. Equation (2) was also independently derived in 1930 by Wood^18^.

The fundamental resonance mode, excited by tapping the stirrer bar against the inner wall of the dissolution vessel is measured using a microphone. The fundamental resonant frequency is determined by the sound velocity in the liquid and the approximate but fixed height of the liquid level, which corresponds to one quarter of its wavelength. The resonant frequency response is explained as;

4 $$freq=\frac{{freq}_{w}}{\sqrt{1+1.49\times {10}^{4}{f}_{a}}}$$

where freq and freq_w_ are the resonance frequencies of the fundamental resonance modes in bubble-filled and pure water, respectively. The total volume of the gas is due to entrained gas, gas due to oversaturation, and gas escaping the solvent due to elimination at the surface or reabsorption. The principles of BARDS analysis is outlined by Fitzpatrick et al.^19^. The acoustic phenomenon used in BARDS has also been demonstrated by Travnicek et al. and several other authors^20–26^.

The aim of this research is to test whether BARDS has the ability to distinguish between genuine and falsified medicines from diverse sources. Different pharmaceutical formulations were analysed. Method development was carried out on falsified and genuine tablet and capsule formulations to obtain a unique signature for genuine formulations. It is shown that the signatures of the falsified formulations significantly differed from the genuine, thus showing the potential of BARDS as a rapid screening tool.

## Experimental

### Instrumentation

**Figure 1:**
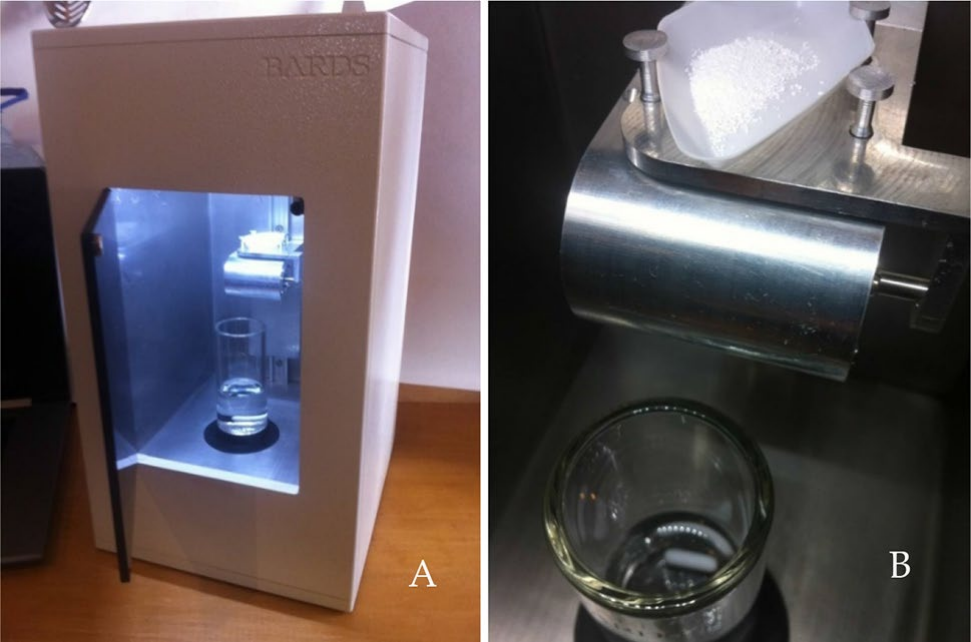
(**A**) External view of the BARDS instrument and (**B**) Tipper motor with a powder sample in a weighing boat ready for addition to the stirred solution below^27^.

A BARDS spectrometer as shown in Fig. 1 was procured from BARDS Acoustic Science Labs (BASL, Bioinnovation Labs, University College Cork). It consists, as described by Ahmed et al., of a chamber with a glass dissolution vessel, a microphone, a magnetic stirrer and a stir bar^27^. There is access at the front to the dissolution vessel and at the top in order to place a sample in a weighing boat on a tipper motor for introduction of the formulation. The microphone is positioned above the top of the glass within the housing. The glass, containing 25 mL of solvent is placed on the stirrer plate. The stirrer motor underneath is positioned so as to allow the magnetic stir bar to gently tap the inner glass wall with stirring rate at 500 rpm. In this way, the stir bar acts as a source of broadband acoustic excitation, thereby inducing various acoustic resonances in the glass, the liquid and the air column above the liquid. The resonances of the liquid vessel are recorded in a frequency band of 0–20 kHz.

#### Material

The following falsified and genuine medicines were received from GlaxoSmithKline plc (GSK); Augmentin (amoxicillin-clavulanic acid) 625 mg tablets, Panadol Extra (paracetamol 500 mg-caffeine 65 mg) tablets, Alli (orlistat) 60 mg capsules, Zentel (albendazole) 200 mg tablets, Clamoxyl (amoxicillin) 500 mg capsules. A set of genuine and falsified artesunate antimalarial tablets were provided by the Medicine Quality Research Group of the Infectious Diseases Data Observatory (IDDO))/MORU Tropical Health Network. Artesunate tablets were a common treatment for *Plasmodium falciparum* malaria when collected but are no longer recommended as first line treatment and have been superseded by co-formulated artemisinin-based combination therapy. Amoxicillin-clavulanic acid coformulation and amoxicillin are commonly used antibiotics, albendazole is used to treat a broad range of parasitic worm infections, paracetamol is an analgesic and orlistat is a weight loss medicine indicated in overweight adults.

The falsified antimalarial samples were purchased in Vietnam in January 2003 (see Table 1), and although were long past the stated expiry date at the time of analysis, they were stored in a fridge and were in apparent excellent condition^28^. They were labelled, falsely, as made by Guilin Pharmaceutical Co. and composed of two packaging types (see “Supporting Information”, Ref.^29^), Type 11 (three samples) and Type 2 (two samples). Genuine tablets labelled as made by Guilin Pharmaceutical Co., of a similar age were also tested as controls.

**Table 1:** Antimalarial tablets, stated as containing 50 mg artesunate labelled as Artesunate and made by Guilin Pharmaceutical Co. Ltd and were obtained in Vietnam^28^.

Sample code and type	Packaging type	Blister batch no.	Blister manufacture date	Blister expiry date
2/15002 Falsified	11	010401	04-2001	04-2004
2/15007 Falsified	11	010401	04-2001	04-2004
2/15013 Falsified	11	010401	04-2001	04-2004
2/15022 Falsified	2	980502	05-1998	05-2002
2/15026 Falsified	2	980502	05-1998	05-2001
G015 Genuine	–	081004	10-2008	10-2011
G050 Genuine	–	010401	04-2001	04-2004
G053 Genuine	–	990801	08-1999	08-2002

The artesunate (stated as 50 mg) tablet samples were received in their original blister packs and stored in plastic bags and were visually identical. Samples were tested by dissolving half tablets in 25 ml of solvent for each BARDS test. Samples were tested initially in deionised water but a clear spectrum for some samples were not achievable due to low solubility. The artesunate samples produced data with greater reproducibility using 0.1 M hydrochloric acid (HCl), which mimics stomach acidity. Thus, 0.1 M HCl was chosen as the test solution medium for BARDS analysis for all artesunate samples. All artesunate samples measurements were carried out in duplicate.

### Experimental procedure

In a typical BARDS analysis, the spectrometer records the steady state resonances of the system as a background reference for 30 s, before the sample is added and the stirrer is set in motion. The pitch of the resonance modes in the solution change significantly when the sample formulation is added before gradually returning to steady state over several minutes^30,31^. The received samples were of different formulations. Therefore, method development was carried out on each formulation in order to find the best method to run the samples. Amoxicillin-clavulanic acid tablets were crushed and 200 mg of powder tested. Amoxicillin powder (200 mg) was removed from the amoxicillin capsules. The tablets of artesunate were cut into halves using a pill cutter obtained from Safe and Sound Health. The entire content of orlistat and amoxicillin capsules were tested for each individual experiment. All samples were tested using 25 ml of solvent of either deionised water or 0.1 M HCl.

## Results and discussion

**Figure 2:**
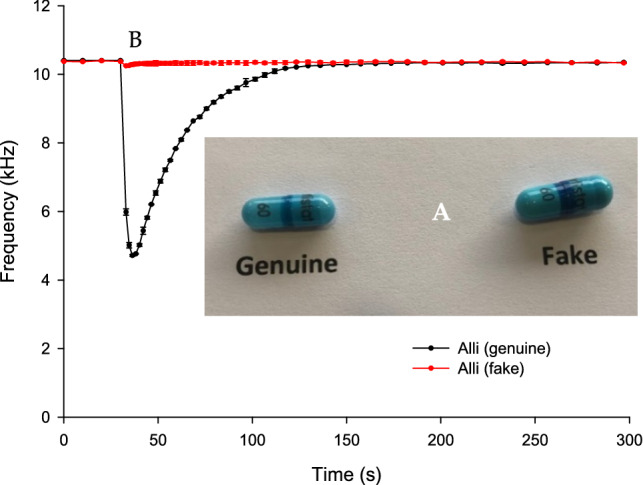
(**A**) an image of genuine and falsified Alli (orlistat) capsules. (**B**) BARDS data of 120 mg of genuine and falsified orlistat powder content dissolved in 25 ml of H_2_O.

Figure 2A shows the visual similarity of genuine and falsified Alli (orlistat) capsules. The only observed visual difference is the dark blue (indigo) band that is thicker in the falsified capsule compared to the genuine one. The genuine capsule weight was 0.18 g and that of the falsified capsule 0.38 g. Also, it was noticed that when attempting to open the capsules, the falsified capsule had a low-quality shell and more easily broke into fragments compared to the genuine capsules. The content of the genuine and the falsified capsules were approximately 0.120 g and 0.250 g, respectively. Figure 2B represent BARDS data of a 120 mg powder sample of genuine and falsified orlistat formulations in deionized water. The dissolution of the whole capsules (shell included) of both genuine and falsified capsules did not yield clear spectra due to the lack of solubility of the shell in water. Thus, only the capsules contents were used for BARDS analysis.

BARDS data clearly differentiates between the genuine and the falsified orlistat as shown Fig. 2B. The resonant frequency of the vessel reduced significantly (*f*_min_ = 4.7 kHz) upon the addition of the genuine capsule content. It then returned to steady state at 129 s, producing a V-shape spectra. The V-shape indicates a rapid evolution of gas followed by slower loss of gas until equilibrium is reached. On the other hand, falsified capsule content produces an insignificant effect on the frequency due to lack of evolution of gas bubbles being produced.

**Figure 3:**
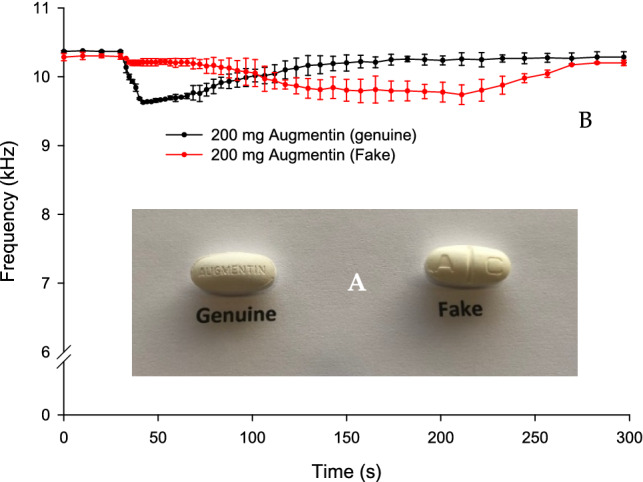
(**A**) An image of genuine and falsified amoxicillin-clavulanic acid tablets. (**B**) BARDS profile of 625 mg of genuine and falsified amoxicillin-clavulanic acid powder dissolved in 25 ml of deionised water.

Figure 3A is an image of the genuine and falsified amoxicillin-clavulanic acid tablets. The shape and size of both tablets were relatively similar, but the recorded weight was ~ 20% different (mean = 1.075 g for the genuine and 1.265 g for the falsified tablet). Both tablets showed different visual characteristic such as the embossed letter on the fake tablet that was different from the genuine one. The falsified tablet also had a score line down the centre. Both tablets were crushed into homogenous powder and 200 mg were dissolved in deionised water. This approach was chosen due to the poor dissolution of the whole or half tablet and also the mass of the tablets/half-tablets was too large to use in a 25 ml BARDS vessel which affected the generated data. The genuine sample produced BARDS spectra (black profile) that can be distinguished from the falsified sample spectra (red profile) in Fig. 3B. The genuine dissolution profile returned to steady state at 157 s while the falsified sample returned to steady state at 282 s. BARDS data is highly reproducible and the average standard deviation across spectral data points of triplicate measurements is ~ 0.122 kHz. This allows for a reduction in replication to duplicate measurements with an average % variance of ~ 2.0%^30,31^. The data of both samples in Fig. 3 were reproducible, however, the falsified sample show slightly larger error bars which may indicates some blend inconsistency of the tablet formulation.

**Figure 4:**
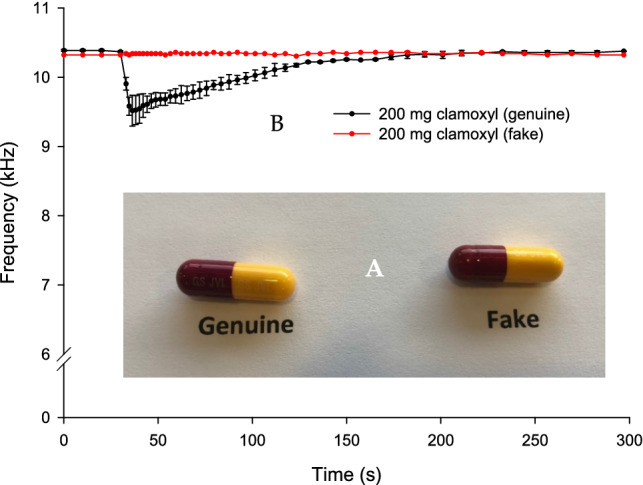
(**A**) An image of genuine and falsified amoxicillin capsules. (**B**) BARDS data of 200 mg of genuine and falsified amoxicillin powder content dissolved in 25 mL of deionised water. Note the falsified data is a single replicate due to the powder being insoluble.

Both genuine and falsified capsules of amoxicillin were alike with almost no visual differences as shown in Fig. 4A. The mean measured weight of falsified and genuine capsules were 0.427 g and 0.700 g, respectively. The powder capsule content was yellowish for the genuine and white for the falsified sample.

BARDS testing of genuine and falsified amoxicillin was performed using 200 mg of capsule powder content and deionised water. Only a single replicate of the falsified powder was attempted. This is due to the capsule content being insoluble and floating on the surface of the solution (deionised water). The BARDS spectra of genuine product produced an *f*_min_ = 9.5 kHz followed by a slow return to steady state at 182 s as shown in Fig. 4B. The falsified amoxicillin powder generated no gas upon its addition to the solvent. Thus, no BARDS spectrum was produced. This behaviour is opposite to that of the genuine product, therefore, producing additional evidence of falsification.

**Figure 5:**
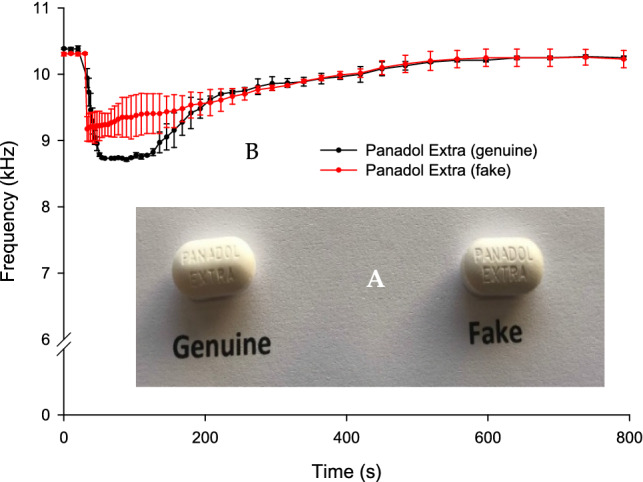
(**A**) An image of genuine and falsified paracetamol/caffeine tablets. (**B**) BARDS data of genuine and falsified paracetamol/caffeine half tablets dissolved in 25 mL of deionised water.

Genuine and falsified paracetamol/caffeine tablets were identical in shape and size, as shown as Fig. 5A, whereas the weight of the genuine tablet was ~ 11% higher (0.69 g) than the weight of the falsified tablet (0.62 g). The font size of the embossed letters on the falsified tablet was slightly larger than that of the genuine tablet.

The black profile in Fig. 5B represents the BARDS profile of genuine tablets whereas the red profile represents the falsified tablet. The *f*_min_ of the genuine sample profile was 8.7 kHz with a plateau for almost 40 s before the start of the return to steady state. However, the falsified tablet BARDS profile begins to return to steady state once *f*_min_ = 9.1 kHz is reached.

**Figure 6:**
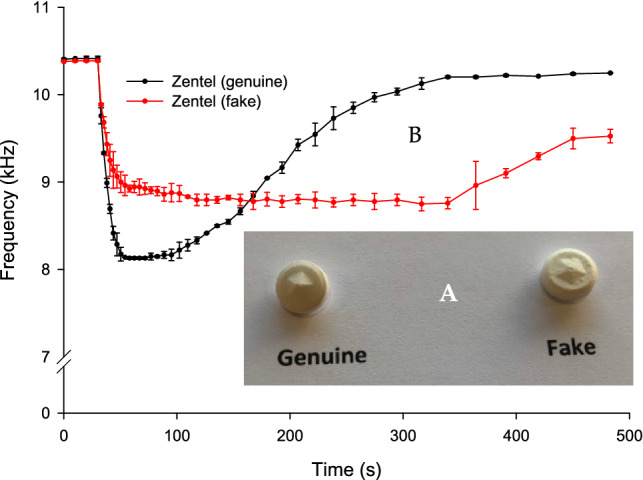
(**A**) An image of genuine and falsified albendazole tablets. (**B**) BARDS data of genuine and falsified half tablets of albendazole dissolved in 25 mL of deionised water.

Figure 6A shows that genuine and falsified tablets of albendazole appeared identical in shape and other visual features and the mean weight for both tablets were almost the same (0.536 g for genuine tablet and 0.535 g for the falsified tablet).

Half tablets were used for each BARDS run of the albendazole. A statistical difference between the BARDS dissolution profiles of the genuine tablets (black profile) and the falsified tablets (red profile) is shown in Fig. 6B. The BARDS profile of the genuine tablet generated an *f*_min_ value of 8.1 kHz and a return to steady state at 339 s with significant reproducibility as indicated by the minor spread in the data. Conversely, the data spread of falsified tablets BARDS profiles are greater than the genuine tablet with *f*_min_ = 8.7 kHz. There is a plateau for approximately 250 s at *f*_min_ with no return to steady state by the end of the experiment.

**Figure 7:**
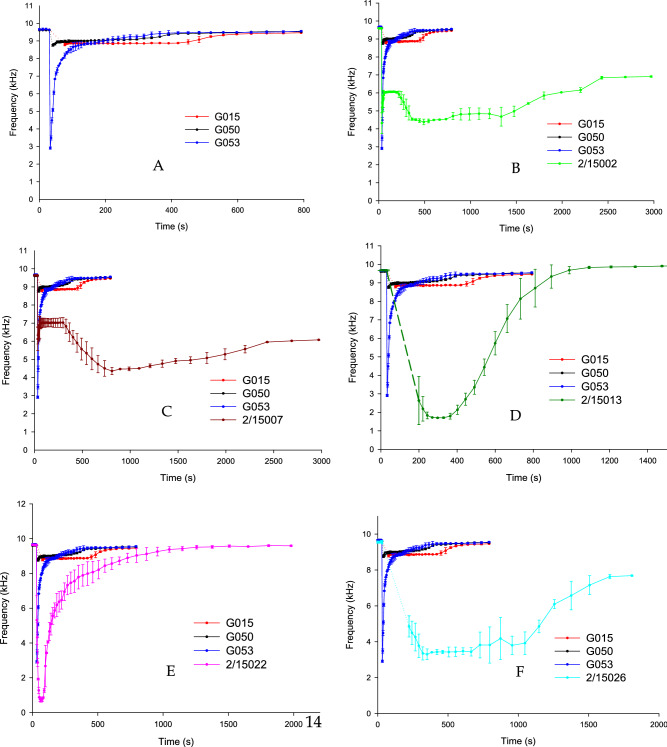
(**A**) BARDS profiles of three batches of genuine artesunate tablets manufactured on different dates, dissolved in 25 ml of 0.10 M HCl. (**B**) BARDS Analysis of falsified (2/15002) and genuine artesunate tablets (G015, G050, G053) dissolved in 25 ml 0.1 M HCl. (**C**) Comparison of falsified artesunate tablets (2/15007) and genuine artesunate tablets (G015, G050, G053) dissolved in 25 ml of 0.10 M HCl. (**D**) Comparison BARDS profiles of falsified artesunate 2/15013 and genuine (G015, G050, G053) artesunate tablets. (**E**) BARDS analysis of falsified (2/15022) and genuine (G015, G050, G053) artesunate tablets dissolved in 25 ml 0.1 M HCl solvent. (**F**) BARDS data of falsified (2/15026) and genuine (G015, G050, G053) artesunate tablets dissolved on 25 ml 0.1 M HCl.

Figure 7A shows BARDS profiles of three different batches of genuine artesunate tablets in 25 ml of 0.1 M HCl. The dotted line on the graphs represents time points where no BARDS signal was observed. Samples G015 and G050 resonant volume lines are absorbed (no longer resonant) after the addition of the samples and became briefly undetectable, however, the spectra became detectable once more at 77 s and 40 s, respectively. There was a significant decrease in frequency, detected for the BARDS profile of sample G053 with an *f*_min_ value = 2.9 kHz. There was good intra-sample correlation indicating good reproducibility.

Figure 7B represent the BARDS profiles of the three genuine artesunate tablets compared to falsified tablet 2/15002 s (green profile). All three genuine tablets returned to steady state before the end of 600 s run time while the BARDS profile of falsified artesunate tablets extended to 3000 s. Upon addition of the falsified artesunate sample at 30 s the frequency dropped to 6 kHz and levels off for approximately 170 s. The genuine samples were analyzed by fluorescence FTIR in October 2015 (unpublished data) shortly before the BARDS analyses. These analyses showed that the three genuine samples contained artesunate with concentration within 90–110% of the stated amount. The bulk excipient for genuine tablets is most likely starch but calcium carbonate for the falsified^29^.

Figure 7C represents comparison between the genuine artesunate BARDS profiles and falsified sample 2/15007. The sample (2/15007) showed a similar behaviour to the falsified sample 2/15002 in Fig. 7B. A lag time at a frequency of 7 kHz lasted for 170 s which may indicate the same coating layer was used similar to sample 2/15002. Sample 2/15007 has an *f*_min_ = 4.3 kHz and the signal levels off by the end of the experiment at 5.9 kHz.

Figure 7D shows the BARDS profile of falsified artesunate tablet (sample 2/15013). The dotted line of the green profile represents time points where no BARDS signal was observed. Consequent to the addition of the tablet at 30 s time point the resonant frequency dropped significantly to *f*_min_ = 1.7 kHz and returned to steady state at 1092 s.

Figure 7E represents the BARDS profile of the genuine artesunate tablets and falsified artesunate tablet sample 2/15022. Once the sample was added to the HCl solution at 30 s the resonant frequency decreased significantly to reach *f*_min_ = 0.7 kHz and returned to steady state at 1270 s. The rapid drops in frequency indicates the disintegration/dissolution of the falsified tablet.

Figure 7F shows the BARDS profile of falsified artesunate tablet 2/15026. The measured weight of the falsified tablet=0.358 g. The BARDS profile of this sample shows large error bars which may indicate inconsistency of the sample formulation. The sample spectra reach an *f*_min_ value = 3.3 kHz and levels off for approximately 300 s. The BARDS profile indicates that high compression force may have been applied to the production of this fake tablet.

Principal Component Analysis (PCA) was used to further analyse the BARDS spectral profiles from the artesunate tablets. The time region from 240 to 740 s was used to compare between all the artesunate tablets with linear interpolation used to ensure direct time point comparison. Figure 9A shows the region of comparison across all the artesunate tablets. Figure 9B shows the PCA score plot for principal component 1 and 2, which together account for more than 99% of the variation between all the tablets. The difference between the profiles is mostly distinguished according to the PC1 score (shown as t1 on the graph). A horizontal dashed grey line is shown in Fig. 9B highlighting where the threshold value for t1 could be set when attempting to rapidly distinguish between genuine and falsified artesunate tablets. Typically, a larger or more representative data sets of genuine artesunate tablets would be required to clearly define such a threshold or distance measure in PCA space.

**Figure 8:**
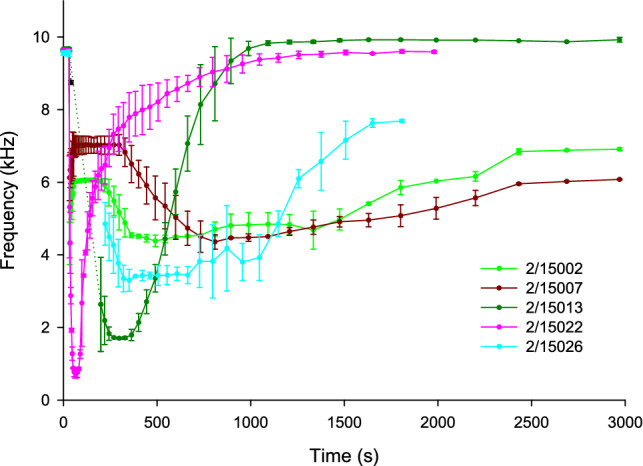
Composite of BARDS profiles of the falsified artesunate tablets.

**Figure 9:**
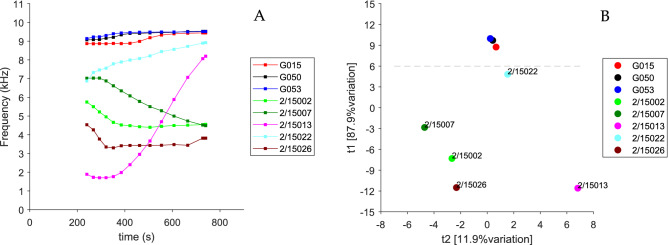
(**A**) BARDS profile time region of comparison across all the artesunate tablets. (**B**) Principle component scores plot for first (t1) and second (t2) principle components, the dashed grey line highlights a potential threshold value to distinguish between genuine and falsified artesunate tablets.

BARDS profile data of artesunate tablets clearly differentiates between the genuine and falsified tablets. None of the falsified samples have similar acoustic profiles to one another or to the genuine product as demonstrated in Figs. 7 and 8 and in Fig. 9 with the use of PCA scores plot. This is conclusive evidence of falsified product detection, whereas the genuine product made 8 years apart, have very similar BARDS profiles and PCA scores. Notably some of the falsified samples also fail to return to steady state by 3000 s.

## Discussion/conclusion

BARDS was shown to differentiate between genuine and falsified samples of diverse medicines. This represents a rapid approach to screen suspect medicines using genuine products as a reference control. BARDS represents a promising new, simple, cost-effective and rapid approach to discriminate between genuine medicines and falsified medicines, taking just 5 min or less. There are no costly reagents or consumables required. A reusable weighing boat can be used. There is no maintenance required apart from daily cleaning. It takes just a few minutes to clean the vessel with water between samples before proceeding with the next sample. An unskilled operator could be trained in less than an hour to process a sample and to identify a falsified product compared to a reference spectra in a database. A simple pass/fail option can also be programmed into the software based on acoustic resonances being present at several time/frequency points which should be present for a genuine product. Alternatively, the spectral profile can be analysed, in part or in full, by multivariate techniques such as PCA, to easily and rapidly differentiate between the genuine and falsified medicines.

BARDS data illustrates that genuine medicines have unique signatures which are not mimicked by fake medicines included in this proof of principle. It does not focus on API content as other techniques do. Instead, it tracks unique disintegration and dissolution processes of the whole tablet or powder. The BARDS response is dependent on several factors including the excipients, the blend ratio, the compression force used to make the tablets and even the speed at which the blend is conveyed during processing^32^. These parameters contribute to the quality attributes of a tablet or lack thereof. The multi-factorial contributions to a BARDS response are responsible for the intrinsic and unique spectrograms which can be harnessed as a tool to differentiate. This sets BARDS apart from other techniques which mainly focus on API identification only. Some formulations are susceptible to degradation for a variety of reasons. These changes could potentially also be tracked. It could also be used to determine formulations from the same source as a presumptive test. Products made by the same manufacturer but marketed under different brand names in different regions also display matching BARDS spectra^33^.

Figure 3B indicates slow disintegration and return to steady state of the amoxicillin-clavulanic acid falsified tablets. This may be due to lack of a disintegrant and/or the excessive use of hydrophobic substances such as magnesium stearate in the powder blend which delays the return to steady state by reducing the rate of water diffusion (wetting)^32^. The same can be said for the profile of the genuine tablet in Fig. 4B. This slow return to steady state is possibly due to the presence of magnesium stearate which is used as a lubricant. Magnesium stearate slows the hydration process, thus delaying the rate of the equilibrium process^32^.

There are now several BARDS studies of genuine formulations which have been cross validated using UV–Vis or HPLC. These studies have shown how coating erosion, disintegration, deaggregation and dissolution can be mapped onto a BARDS spectrum. The UV–Vis data shows that ~ 50% of drug is released by the time the BARDS minimum is reached and the remainder as the frequency returns to steady state^15^.

In Fig. 6B there is a plateau for approximately 250 s at *f*_min_ with no return to steady state by the end of the experiment for the falsified albendazole. This may be an indication of excessive compression force being used in the production of these falsified tablets, leading to prolonged disintegration and less trapped air as indicated by the increased value of *f*_min_. Also, the use of hydrophobic materials can affect the return to steady state frequency. Conversely, powders that have faster wetting rates have a faster return to steady state^30,32^. There tends to be less disintegrant used in falsified tablets compared to genuine.

The data spread of the falsified paracetamol/caffeine sample in Fig. 5 was larger than those of the genuine tablets. This may indicate that the blend uniformity is less than that of the genuine tablet. The increased *f*_min_ value for the falsified tablet may also indicate an increased compression force was used to make the tablet which leads to less voids between particles, thus less trapped air^33^.

The relatively slight variation in spectra between genuine artesunate in Fig. 7A may be due to inter-batch variation or different formulation and excipient grades being used. G050 and G053 samples returned to steady state at almost the same time while G015 took longer to disintegrate with later return to steady state. This could be due to the slight variation in compression force used in production. Also, the stability of the three samples and the wide range of manufacture and expiry dates could have caused BARDS profile variation. G015, G050 and G053 were manufactured in 2008, 2001 and 1999, and had expiry dates of 2011, 2004 and 2002, respectively.

The plateau in Fig. 7B may indicate that some coating layer may have been added to the falsified artesunate. Also, the BARDS profiles suggest that a larger compression force applied during the manufacturing process of these falsified tablets. This may explain the prolonged disintegration of this tablet during BARDS tests, which lasted for 3000 s with no return to steady state frequency. High compression force would reduce the voids between particles which leads to longer hydration process times. Also, poor wettability of substances used in this formulation could have caused the delay observed in the BARDS profile.

Although similar, the BARDS profile of the falsified artesunate in Fig. 7C is different from the falsified sample in Fig. 7B. These differences may also be due to different compression forces being applied in the production of the tablets as well as different formulation processes and materials being used.

The BARDS profile of the falsified tablet in Fig. 7D yielded a U-shape spectra. This may be a result of using a greater mass of sample. This is confirmed by the measured weight of the whole tablet which was 0.334 g while the artesunate genuine tablets weight was approximately 0.270 g.

Generally, varying frequency minima in the spectra, as well as the time taken to return to steady state of the system are all indicators as to whether the material being tested is authentic or falsified when compared to the genuine medicines. The PCA analysis employed for the artesunate tablets in Fig. 9B also showed how the BARDS profile can be analysed to easily compare between tablets. The genuine tablets were distinguished by a high score value in t1. Falsified tablets could be immediately distinguished by a low or negative score in t1 as well as lager values in t2. The analysis suggests some potential similarity between falsified tablets from 2/15007, 2/15002, 2/15026 whereas tablets from 2/15013 and 2/15022, while also falsified, were clearly different in their score values and profile response from the other falsified tablets. The ability to easily identify differences and similarities between falsified tablets may be a desirable output of multivariate BARDS data analysis. With the availability of larger or more representative data sets of tablets or capsules, thresholds and distance measure in PCA space can also be used to clearly identify acceptable variation for genuine tablets or capsules, making them readily distinguishable from their falsified counterparts.

There was no correlation found between the artesunate packaging type and their BARDS spectra. Substandard medicines, both due to within-factory errors and due to degradation in supply chains, are expected to produce different BARDS spectra compared to control spectra with the correct formulation. Small percentage changes of the excipients can cause statistical changes in the spectra^34^.

Future work will investigate a broader range of pharmaceutical products with different quality profiles to produce a larger statistical dataset of genuine and falsified medicines in an on-line reference library.

Currently, BARDS is a fully portable laboratory benchtop device (about a quarter of the price of a HPLC system). Like Raman or near-infrared spectrometers, BARDS could be easily adapted for field testing with a battery. It could provide on the spot testing of suspect products coupled with cloud internet access in remote areas to a spectral database in post-market surveillance for the objective selection of samples for formal reference laboratory analysis. One limitation of BARDS is the need for authentic reference signatures for the wide diversity of brands available on the market. There are currently ~ 7000 international non-proprietary names (INN) of pharmaceutical substances globally, and probably hundreds of thousands of brands. However, it would be an interesting device for targeted INN/brands, in risk-based post marketing surveillance. (https://www.who.int/teams/health-product-and-policy-standards/inn/guidance-on-inn).

Limitations of the study include that only a few API were tested, out of the many thousands in use globally, and we did not include fixed-dose co-formulated medicines. We did not examine the influence of batch to batch and brand variation and degradation on acoustic profiles. Optimisation of solvent used for diverse APIs and understanding of the appropriateness of the technique for fat-soluble drugs remains to be determined.

Although a neglected area of research, many of those who have investigated medicine disintegration and dissolution issues, have found evidence of major impairments. In recent surveys in Afghanistan, 58/203 antimalarials failed disintegration tests and in the Democratic Republic of Congo, 5/20 antimalarial quinine tablets failed dissolution tests^35,36^. Disintegration tests are often used in screening, such as that available in the portable GPHF-Minilab, but are infrequently followed by laboratory-based dissolution tests. Traditional disintegration only tests part of the process of drug release and has poor prediction to identify samples with extreme dissolution failure^37^. The disintegration testing in the GPHF-Minilab kit is thus not an appropriate proxy for dissolution testing. Only two of the 25 antimalarial samples that failed dissolution testing failed the GPHF-Minilab disintegration test. The portfolio of medicine quality screening devices would greatly benefit from reliable portable screening device for assaying dissolution. Although more investigations are needed, BARDs looks-like a promising candidate for this purpose.
